# New Evidence for the Theory of Chromosome Organization by Repetitive Elements (CORE)

**DOI:** 10.3390/genes8020081

**Published:** 2017-02-20

**Authors:** Shao-Jun Tang

**Affiliations:** Department of Neuroscience and Cell Biology, University of Texas Medical Branch, Galveston, TX 77555, USA; shtang@utmb.edu; Tel: +1-409-772-1190

**Keywords:** chromosome, chromatin, repetitive DNA, CORE theory, chromosome conformation capture

## Abstract

Repetitive DNA elements were proposed to coordinate chromatin folding and interaction in chromosomes by their intrinsic homology-based clustering ability. A recent analysis of the data sets from chromosome-conformation-capture experiments confirms the spatial clustering of DNA repeats of the same family in the nuclear space, and thus provides strong new support for the CORE theory.

Chromatins are the longest molecular complexes that store genetic information in eukaryotic cells, and they must be highly folded and packaged into chromosomes to fit in the tiny nuclear space. How chromatins are orderly folded in chromosomes remains a fascinating and fundamental mystery in biology. During cell cycles, a specific chromatin of a given species (e.g., human) is always folded into a metaphase chromosome with a similar shape. This observation indicates that chromatin folding in the cells is an accurate process with a high reproducibility. Yet, the mechanism that ensures such a high reproducibility of chromosome organization is unclear. In the past decades, the mainstream effort of searching for the molecular players in chromosome packaging has been concentrated on identifying potential packaging proteins. However, the high fidelity of chromatin folding during chromosome packaging apparently suggests that the DNA in chromatins also contains critical information that directs chromatin folding. Interestingly, the identification of potential DNA elements dedicated to chromosome packaging has never been the focal point of research. Recently, the analysis of multiple lines of evidence leads to the conclusions that chromatins with repetitive DNA elements, which are often thought of as genomic junks (“junk DNA”), have the intrinsic property to interact with their homologous regions in chromosomes and that the interaction drives the folding of chromatins in a repeat-guided fashion in the cell [1]. This proposal, called the CORE theory (Chromosome Organization by Repetitive Elements), appears to provide a simple mechanistic insight into the formation of higher-order chromosome organization. Guided by this theory and available data on the spatial organization of repetitive DNA in chromosomes, specific models of mitotic and interphase chromosomes have been put forward [2,3]. The key idea of the CORE theory is that association of homologous repeats in the nucleus creates a driving force for chromosome organization. This theory leads to a specific prediction of spatial clustering of dispersed repeat elements in the nucleus. Although the idea of spatial clustering of similar repeats is consistent with various studies that used different approaches, such as fluorescent in situ hybridization (FISH) (references in [1]), a recent study by Cournac et al. [4] provides a systematic and conclusive test of this prediction.

Cournac et al. [4] used computational approaches to analyze the potential spatial co-localization of similar repeats in the nucleus. The authors performed a systematic analysis of published data sets collected from chromosome conformation capture experiments to calculate the co-localization tendency of members in multiple repeat families in three different metazoan genomes. This large-scale analysis offers several advantages for systematically measuring the clustering state of similar repeats, including monitoring multiple repeat subfamilies and comparing the repeat clustering in different cell types and different species. These features of this analysis allow one to conceive a global picture of the spatial patterns of repeats in chromosomes.

The authors first determined the co-localization score (CS) of multiple repeat families in human embryonic stem cells (hESC), including satellites/low complexity sequences, short interspersed elements (SINE), long interspersed elements (LINE), long terminal repeats (LTR), DNA transposons and RNA repeats. They found that all families have subfamilies with significantly higher CSs. This finding suggests that spatial clustering in the nucleus is a general property of repeated elements in human cells. Overall, similar results were also obtained from two other human cell lines, although some local changes were observed. In addition, the authors observed that repeats with significant co-localization scores tended to be the evolutionarily older ones, indicating that spatial association with similar repeats is a selected property of repeats during evolution.

Furthermore, the authors also computed repeat co-localization in other metazoans, including mouse embryonic stem cells (mESC) and *Drosophila* embryos. Similar to human cells, repeats in mESC also showed trends of co-localization. Although some repeats found in mammalian genomes are not in *Drosophila*, the repeats that are found in both organisms do show significant tendency of three-dimensional clustering. These findings strengthen the idea of spatial self-clustering as an evolutionarily conserved behavior of repetitive elements.

Thus, the findings from the Cournac et al. study add a new set of systematic evidence for the spatial clustering of homologous repeats in different subfamilies in the nucleus. Although these findings are consistent with the key prediction of the CORE theory, they do not by themselves indicate whether the clustering is an active causal driver for or a passive consequence of chromatin folding. In this context, it is relevant to point out that, similar to the self-assembly of DNA [5], recent studies show that chromatins with homologous DNA also exhibit the ability to self-assemble [6,7,8]. These observations suggest the intrinsic property of homology recognition in chromatins.

Results from the Cournac et al. study add strong support for the conclusion that repetitive DNA elements, in general, have the intrinsic tendency of self-assembling in vivo [1]. As self-assembling of repeats in linear genomes will inevitably lead to chromatin folding, their work further supports the CORE theory. Under the perspective of CORE, repetitive DNA elements function as the structural code for chromosome organization in the eukaryotic cells [1]. The distribution pattern of repetitive DNA elements along the linear chromatin contains the genetic information that dictates chromatin folding during chromosome packaging. Future research on the role of repetitive DNA elements in chromosome packaging is expected to be fruitful and may lead to the discovery of genetic blueprints of higher-order chromosome organization in the genomes.
